# Development of machine learning-based predictors for early diagnosis of hepatocellular carcinoma

**DOI:** 10.1038/s41598-024-51265-7

**Published:** 2024-03-04

**Authors:** Zi-Mei Zhang, Yuting Huang, Guanghao Liu, Wenqi Yu, Qingsong Xie, Zixi Chen, Guanda Huang, Jinfen Wei, Haibo Zhang, Dong Chen, Hongli Du

**Affiliations:** 1 School of Biology and Biological Engineering, South China University of Technology, Guangzhou, China; 2 Key Laboratory of Ministry of Education for Gastrointestinal Cancer, School of Basic Medical Sciences, Fujian Medical University, Fuzhou, 350122 China; 3 Fujian Key Laboratory of Medical Bioinformatics, Department of Bioinformatics, School of Medical Technology and Engineering, Fujian Medical University, Fuzhou, 350122 China; 4 Fangrui Institute of Innovative Drugs, South China University of Technology, Guangzhou, China

**Keywords:** Cancer, Computational biology and bioinformatics, Biomarkers, Gastroenterology

## Abstract

Hepatocellular carcinoma (HCC) remains a formidable malignancy that significantly impacts human health, and the early diagnosis of HCC holds paramount importance. Therefore, it is imperative to develop an efficacious signature for the early diagnosis of HCC. In this study, we aimed to develop early HCC predictors (eHCC-pred) using machine learning-based methods and compare their performance with existing methods. The enhancements and advancements of eHCC-pred encompassed the following: (i) utilization of a substantial number of samples, including an increased representation of cirrhosis tissues without HCC (CwoHCC) samples for model training and augmented numbers of HCC and CwoHCC samples for model validation; (ii) incorporation of two feature selection methods, namely minimum redundancy maximum relevance and maximum relevance maximum distance, along with the inclusion of eight machine learning-based methods; (iii) improvement in the accuracy of early HCC identification, elevating it from 78.15 to 97% using identical independent datasets; and (iv) establishment of a user-friendly web server. The eHCC-pred is freely accessible at http://www.dulab.com.cn/eHCC-pred/. Our approach, eHCC-pred, is anticipated to be robustly employed at the individual level for facilitating early HCC diagnosis in clinical practice, surpassing currently available state-of-the-art techniques.

## Introduction

Worldwide, liver cancer is the fourth most common cause of death from cancer according to the 2021 Global Cancer Statistics Report^1^. Approximately 90% of liver tumor cases are HCC patients. Cirrhosis of the liver from any cause is the most serious risk factor for HCC^2,3^, as over 80% of HCC are developed from liver on a cirrhotic background. Generally, HCC can be diagnosed either based on imaging or by biopsy. However, imaging criteria for HCC diagnosis are only applicable to high-risk patients, comprising those with chronic HBV infection or cirrhosis. Additionally, imaging is difficult to determine whether lesions with diameter of < 1 cm are HCC or not. Although most HCC patients have characteristic imaging manifestation, about 10% of tumors (up to 30% of tumors with diameters of 1–2 cm) lack imaging features of HCC^4^. If HCC is suspected clinically but imaging findings are atypical, a biopsy or second examination should not be delayed, if the second examination is inconclusive, a biopsy is performed^3^. Whereas, biopsy may lead to misdiagnosis (false-negative results) when biopsy samples were obtained from inaccurate locations. For inaccurately sampled HCC biopsy specimens, with adjacent non-tumor (cirrhosis or normal) tissues, the diagnostic false-negative rate of small biopsy specimens is approximately 30–50%^5,6^. Thus, it is essential to design novel molecular signatures for diagnosis of early HCC, particularly when the locations of biopsy samples are inaccurate.

Over the past several years, based on gene expression profiles, different signatures for the early diagnosis of HCC have been proposed by researchers. Since within-sample relative expression orderings (REOs) of genes is less sensitive to experimental batch effects, qualitative transcriptional signatures constructed by REOs can be utilized to samples at an individual level^7–11^. Meanwhile, REOs is also robust to RNA degradation during specimen preparation and storage^12^. Some previous work adopted REOs to develop diagnostic marker of HCC^13,14^, gastric carcinoma^15^, colorectal carcinoma^16^, pancreatic ductal adenocarcinoma^10,11,17^ and so on. Thus, it is credible to identify a REOs-based transcriptional signature for early diagnosis of HCC. Nevertheless, it is not yet possible to implement these existing gene signatures in clinical practice even though they have a powerful diagnostic ability for early HCC. That’s partially because these signatures were obtained from gene expression profiling data, which may not provide an accurate reflection of the changes in plasma proteins^18–20^. Since secreted genes can be translated into secreted proteins, which can be possibly used as tumor microenvironment or plasma signatures, we employed secreted genes for filtering feature.

Motivated by the establishment of various diagnostic signatures based on REOs to aid clinical HCC diagnosis decision, we designed robust and powerful predictors in this work. The developed predictors hybridized several algorithms, i.e., REOs, mRMR^21^, MRMD^22^, support vector machine (SVM)^23,24^, k-nearest neighbor (KNN)^24^, decision tree (DT)^25,26^, logistic regression (LR)^26^, extreme gradient boosting (XGBoost)^24^, logistic model trees (LMT)^27^, adaptive boosting M1 (AdaBoostM1)^28^ and naïve bayes (NB)^29^. The REOs method was used for feature construction, mRMR and MRMD were used for feature ranking and selection, 2902 secreted genes (genes encoding secreted proteins) collected public database were used for feature filtering, and SVM, KNN, DT, LR, XGBoost, LMT, AdaBoostM1 and NB algorithms were used for classification purposes. Among the sixteen predictors, nine predictors (including mRMR + KNN, mRMR + SVM, mRMR + LR, mRMR + XGBoost, mRMR + LMT, MRMD + KNN, MRMD + SVM, MRMD + LR and MRMD + LMT) showed excellent results for all performance metrices in training set, and reached accuracy of 1, F1-score of 1 and AUC of 1, respectively. In validation datasets, the AUC value of mRMR + SVM predictor with the least number of 11 gene pairs (AUC = 0.9384) and MRMD + SVM predictor with 28 gene pairs (AUC = 0.9278) were higher among these nine predictors, and they were powerful predictors for HCC diagnosis even when the sampling location is not accurate. Simultaneously, mRMR + SVM predictor and MRMD + SVM predictor had a cross-platform effect and could be employed to diagnose early HCC at individual level. In addition, comparison results demonstrated that the performance of the established hybrid predictor mRMR + SVM and MRMD + SVM were much better when compared with Ao’s method^14^ and our previous work^13^. Importantly, a user-friendly web server was established, and it could be freely accessed at http://www.dulab.com.cn/eHCC-pred/ for aiding the early HCC diagnosis in clinical practice.

## Results

### Derivation of HCC predictors

**Figure 1:**
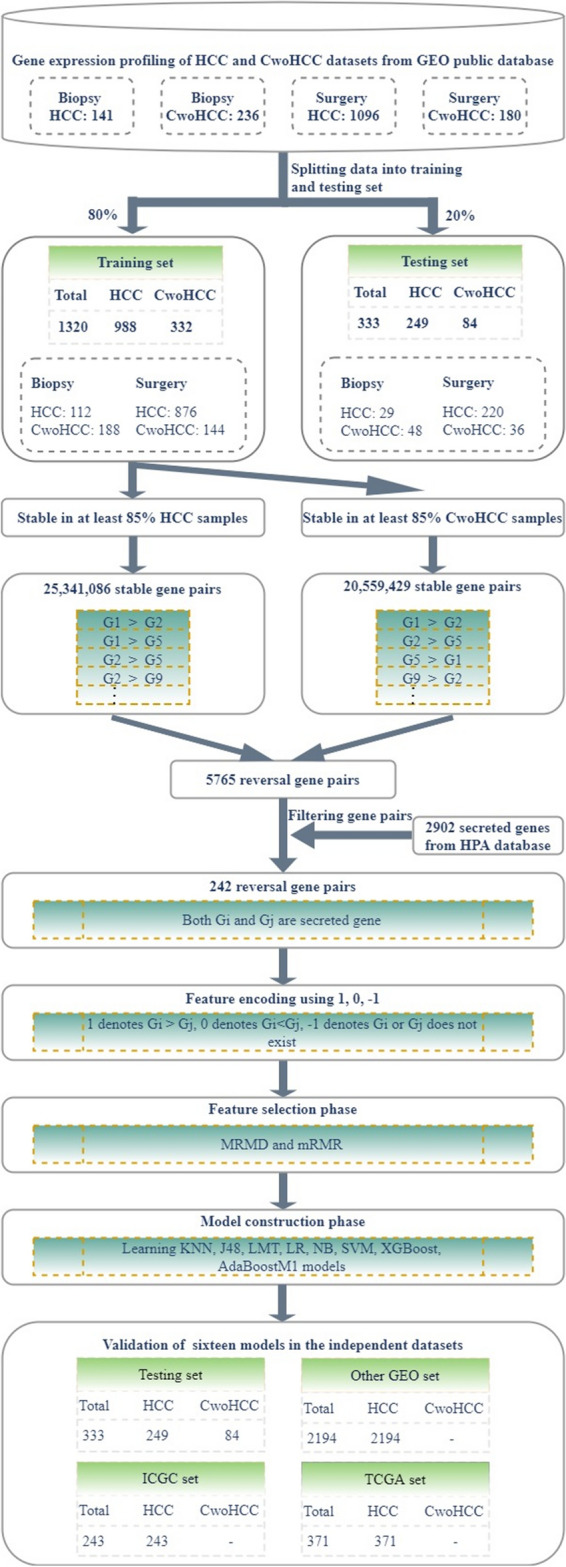
The workflow of analyses.

**Figure 2:**
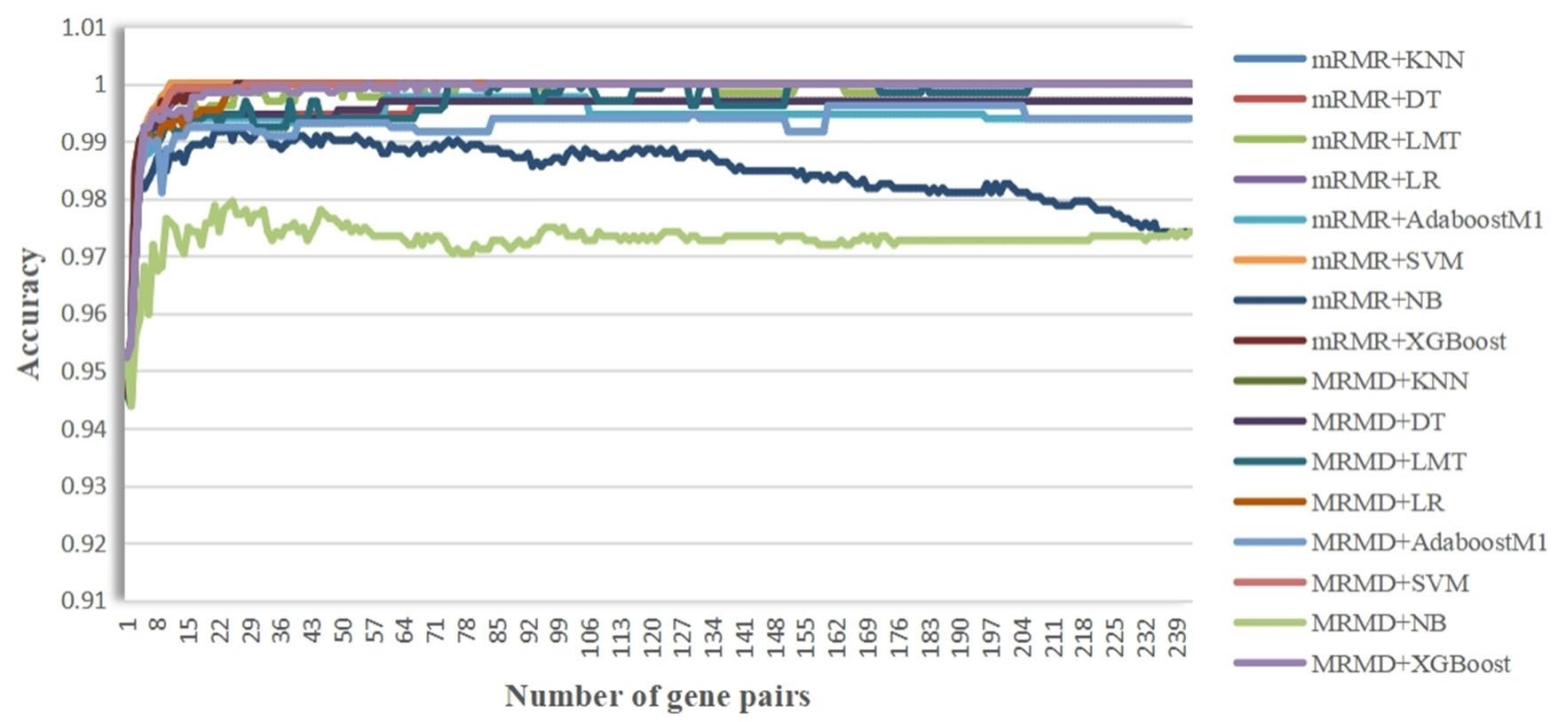
A plot to show the IFS curve. Through adding features (gene pairs) ranked by mRMR and MRMD feature selection method one by one, the optimal feature was obtained when the highest accuracy was achieved.

The whole procedure of analysis was designed as follows in Fig. 1. In present study, we used two feature selection methods and eight classification algorithms mentioned above to build sixteen predictors for HCC diagnosis by using gene expression profiles of 988 HCC and 332 CwoHCC accessed from the GEO database. First, on the basis of gene expression profiles of 988 HCC and 332 CwoHCC, 25,341,086 and 20,559,429 stable gene pairs were acquired, respectively. Among 25,341,086 and 20,559,429 gene pairs, there were 5765 stable reversal gene pairs between HCC tissues and CwoHCC tissues. Then, filtering gene pairs using 2902 secreted genes, we obtained 242 gene pairs, where gene i and gene j were secreted gene. Next, based on novel profiles with 242 features (gene pairs) (see “Methods” section), we captured the optimal feature (see Fig. 2). Table 1 showed the comparison of classification performance of various predictors obtained based on accuracy, F1-Score fitness function and AUC value. The results presented in Table 1 illustrated that nine predictors, including mRMR + KNN, mRMR + SVM, mRMR + LR, mRMR + XGBoost, mRMR + LMT, MRMD + KNN, MRMD + SVM, MRMD + LR and MRMD + LMT, showed excellent results for all performance metrices, and reached accuracy of 1, F1-score of 1 and AUC of 1, respectively. Among these nine predictors, the predictor of mRMR + KNN and mRMR + SVM had the least number of 11 gene pairs (see Table 2).

**Table 1:** Comparison of various predictors based on accuracy and F1-score fitness function with feature selection in training set.

Predictors	NO.Opt	NO.HCC	NO.CwoHCC	ACC	F1-score	AUC	95% CI
mRMR + KNN	11	988/988	332/332	1	1	1	1–1
mRMR + SVM	11	988/988	332/332	1	1	1	1–1
mRMR + LR	15	988/988	332/332	1	1	1	1–1
mRMR + XGBoost	26	988/988	332/332	1	1	1	1–1
mRMR + LMT	26	988/988	332/332	1	1	1	1–1
mRMR + AdaboostM1	60	987/988	330/332	0.9977	0.9985	0.9965	0.9922–1
mRMR + J48	66	987/988	329/332	0.997	0.998	0.995	0.9898–1
mRMR + NB	24	980/988	330/332	0.9924	0.9949	0.9929	0.9879–0.998
MRMD + KNN	28	988/988	332/332	1	1	1	1–1
MRMD + SVM	28	988/988	332/332	1	1	1	1–1
MRMD + LR	30	988/988	332/332	1	1	1	1–1
MRMD + LMT	74	988/988	332/332	1	1	1	1–1
MRMD + J48	59	987/988	329/332	0.997	0.998	0.995	0.9898–1
MRMD + AdaboostM1	160	985/988	330/332	0.9962	0.9975	0.9955	0.991–1
MRMD + XGBoost	96	982/988	326/332	0.9909	0.9939	0.9879	0.9804–0.9955
MRMD + NB	28	963/988	328/332	0.978	0.9852	0.9813	0.9737–0.989

*NO.Opt* number of optimal signature, *NO.HCC* number of HCC samples, *NO.CwoHCC* number of CwoHCC samples, *ACC* accuracy.

**Table 2:** The 11 gene pairs’ signature ranked by mRMR.

Order	Feature (gene pair)	
	Gene i	Gene j
1	PCOLCE2	DBH
2	RPLP2	FCN3
3	THY1	DPT
4	GDF15	CHST4
5	PTPRA	DBH
6	RPLP2	ADAMTSL2
7	PPIC	C7
8	EIF2AK1	F8
9	KDSR	FCN2
10	PRDX4	C7
11	KDSR	ASAH1

Gene i has a higher expression level than Gene j in HCC patients compared with CwoHCC patients.

### Validation of HCC predictors

Subsequently, we used independent datasets (including testing set, GEO sets, ICGC set and TCGA set) to validate the performance of various algorithms. In Table 3, for the 3057 HCC samples and 84 CwoHCC samples, MRMD + SVM predictor with 28 gene pairs (see Table S3) gained the highest accuracy and F1-score than other predictors in independent datasets, the accuracy, F1-score, and AUC were 0.9834, 0.9915, 0.9278 (95% CI is 0.8915–0.9642), respectively. However, the results also indicated that mRMR + SVM predictor with 11 gene pairs gained the highest AUC than other predictors in independent datasets, the AUC was 0.9384 (95% CI 0.9255–0.9514).

**Table 3:** The performance of various predictors in independent datasets.

Predictors	NO.Opt	NO.HCC	NO.CwoHCC	ACC	F1-score	AUC	95% CI
mRMR + KNN	11	2383/3057	83/84	0.7851	0.8759	0.8838	0.87–0.8976
mRMR + SVM	11	2717/3057	83/84	0.8914	0.941	0.9384	0.9255–0.9514
mRMR + LR	15	2155/3057	83/84	0.7125	0.8268	0.8465	0.8323–0.8607
mRMR + XGBoost	26	2204/3057	83/84	0.7281	0.8377	0.8545	0.8404–0.8687
mRMR + LMT	26	2078/3057	82/84	0.6877	0.809	0.828	0.8096–0.8463
mRMR + AdaboostM1	60	2213/3057	83/84	0.731	0.8397	0.856	0.8419–0.8701
mRMR + J48	66	2023/3057	84/84	0.6708	0.7965	0.8627	0.8487–0.8767
mRMR + NB	24	2254/3057	83/84	0.744	0.8486	0.8309	0.8225–0.8393
MRMD + KNN	28	2334/3057	83/84	0.7695	0.8657	0.8758	0.8619–0.8897
MRMD + SVM	28	3016/3057	73/84	0.9834	0.9915	0.9278	0.8915–0.9642
MRMD + LR	30	2337/3057	83/84	0.7705	0.8664	0.8301	0.8117–0.8485
MRMD + XGBoost	56	2508/3057	82/84	0.8274	0.901	0.8983	0.8805–0.9161
MRMD + LMT	74	2285/3057	84/84	0.7542	0.8555	0.8763	0.8624–0.8902
MRMD + J48	59	2297/3057	83/84	0.7577	0.8579	0.8697	0.8558–0.8837
MRMD + AdaboostM1	160	2412/3057	82/84	0.794	0.8817	0.8737	0.866–0.8814
MRMD + NB	28	2091/3057	82/84	0.6918	0.812	0.8826	0.8647–0.9005

*NO.Opt* number of optimal signature, *NO.HCC* samples, number of HCC samples, *NO.CwoHCC* samples, number of CwoHCC samples, *ACC* accuracy.

Since mRMR + SVM predictor and mRMR + KNN predictor with the least number of 11 gene pairs showed great results for all performance metrices in independent data, and MRMD + SVM predictor gained the highest accuracy and F1-score in independent datasets among 16 predictors, thus we focused on these three predictors in the next analysis. The detailed validation results of these three predictors in biopsy and surgery samples were shown in Table 4. For biopsy samples, both mRMR + SVM predictor and mRMR + KNN predictor yielded sensitivity of 1, specificity of 1 by using testing set (29 HCC samples and 48 CwoHCC samples), while MRMD + SVM predictor yielded sensitivity of 1, specificity of 0.8542. In GEO biopsy sets, mRMR + SVM predictor correctly classified 96.18% of the 131 HCC samples (GSE121248, GSE47197), mRMR + KNN predictor correctly classified 66.41% of the 131 HCC samples as well as all (100%) of the 131 HCC samples were correctly classified by MRMD + SVM predictor. For surgery samples, in the testing set (220 HCC samples and 36 CwoHCC samples), the sensitivity and specificity of two predictors (mRMR + SVM predictor and mRMR + KNN predictor) were 1. While, the sensitivity and specificity of MRMD + SVM predictor was 1 and 0.8889. This result demonstrated that mRMR + SVM predictor, mRMR + KNN predictor and MRMD + SVM predictor could discriminate HCC from CwoHCC correctly when using biopsy samples.

**Table 4:** The performance of the 11 gene pairs’ signature in independent datasets.

Dataset	NO.HCC	NO.CwoHCC	mRMR + KNN		mRMR + SVM		MRMD + SVM	
			Sn	Sp	Sn	Sp	Sn	Sp
Testing set (biopsy)	29	48	1	1	1	1	1	0.8542
Testing set (surgery)	220	36	1	1	1	1	1	0.8889
GEO (biopsy)								
GSE121248	70	–	0.9286	–	0.9429	–	1	–
GSE47197	61	–	0.3607	–	0.9836	–	1	–
GEO (surgery)								
GSE109211	140	–	0.7214	–	0.7786	–	0.9929	–
GSE62743	132	–	0.6288	–	0.8636	–	1	–
GSE46444	88	–	0.3409	–	0.5227	–	1	–
GSE10141	80	–	0	–	0.9875	–	1	–
GSE164760	53	–	0.0755	–	0.2453	–	1	–
GSE19977	164	–	1	–	0.9939	–	1	–
GSE112790	183	–	0.9836	–	0.9945	–	1	–
GSE102079	152	–	0.9737	–	0.9934	–	1	–
GSE76427	115	–	0.7826	–	0.9478	–	1	–
GSE78737	103	–	0.2427	–	0.3301	–	0.8544	–
GSE9843	91	–	0.9231	–	0.9231	–	1	–
GSE43619	88	–	0.7273	–	0.8523	–	1	–
GSE62232	81	–	0.9506	–	0.9753	–	1	–
GSE39791	72	–	1	–	1	–	1	–
GSE15765	70	–	0.9571	–	1	–	1	–
GSE87630	64	–	0	–	1	–	1	–
GSE36411	42	–	0.8095	–	0.881	–	1	–
GSE89377	40	–	0	–	0.2	–	0.825	–
GSE57957	39	–	1	–	0.9744	–	1	–
GSE14323	38	–	0.0789	–	0.1579	–	0.5263	–
GSE6764	35	–	0.8	–	0.8286	–	1	–
GSE101685	24	–	1	–	1	–	1	–
GSE84598	22	–	1	–	1	–	1	–
GSE41804	20	–	0.9	–	1	–	1	–
GSE17548	17	–	0.7059	–	0.7059	–	1	–
GSE84402	13	–	0	–	0.0769	–	1	–
GSE115018	12	–	0	–	0.9167	–	1	–
GSE98383	11	–	0.9091	–	1	–	1	–
GSE29721	10	–	0.8	–	0.8	–	1	–
GSE116174	64	–	0.9063	–	1	–	1	–
ICGC (surgery)	243	–	–	–	–	–	–	–
TCGA (surgery)	371	–	–	–	–	–	–	–

*NO.HCC*, number of HCC samples, *NO.CwoHCC*, number of CwoHCC samples, *Sn* sensitivity, *Sp* specificity.

For surgery samples, in GEO surgery sets, 84.1% of the 2063 HCC samples were correctly classified by mRMR + SVM predictor, 70.04% of the 2063 HCC samples were correctly classified by mRMR + KNN predictor and 98.01% of the 2063 HCC samples were correctly classified by MRMD + SVM predictor. Moreover, among 2063 HCC samples, based on mRMR + SVM predictor, 79.76% of the 657 formalin-fixed paraffin-embedded (FFPE) HCC samples (GSE109211, GSE62743, GSE46444, GSE10141, GSE164760, GSE19977) were correctly recognized as HCC; while 58.14% of the 657 FFPE HCC samples was correctly classified by mRMR + KNN predictor and 99.85% of the 657 FFPEHCC samples was correctly classified by MRMD + SVM predictor. This result demonstrated that mRMR + SVM and mRMR + KNN predictor were available to the FFPE samples with RNA degradation. For the RNA-seq expression data obtained from TCGA and ICGC, the 11 gene pairs based on mRMR + SVM predictor could correctly identify 99.19% of the 371 HCC and the 98.77% of the 243 HCC samples, respectively.

While the 11 gene pairs based mRMR + KNN predictor could correctly identify 98.11% of the 371 HCC RNA-seq and the 97.94% of the 243 HCC RNA-seq samples. And MRMD + SVM predictor with 28 gene pairs could correctly identify all 371 HCC RNA-seq and all 243 HCC RNA-seq samples. This result demonstrated that mRMR + SVM predictor, mRMR + KNN predictor and MRMD + SVM predictor had a cross-platform ability. In summary, these three predictors had a cross-platform ability and could discriminate HCC from CwoHCC when using surgery samples, including FFPE samples with RNA degradation.

Furthermore, in Table S4, 82.86% of the 741 normal tissues in patients with HCC samples (NwHCC) samples and 82.04% of the 334 cirrhosis tissues in patients with HCC samples (CwHCC) samples were correctly classified by mRMR + SVM predictor, 67.48% of the 741 NwHCC samples and 57.49% of the 334 CwHCC samples were correctly classified by mRMR + KNN predictor, and 99.87% of the 741 NwHCC samples and 97.01% of the 334 CwHCC samples were correctly classified by MRMD + SVM predictor. This result showed that these three predictors could identify HCC adjacent tissues (CwHCC and NwHCC) from CwoHCC when using biopsy and surgery samples.

In conclusion, for biopsy and surgery samples, these three predictors could identify HCC and its adjacent tissues (CwHCC and NwHCC) from CwoHCC even when sample location is not accurate and samples are FFPE samples with RNA degradation. Additionally, these three predictors had a cross-platform ability. Importantly, the performance of HCC diagnostic signature based on MRMD + SVM is superior to mRMR + KNN predictor and mRMR + SVM predictor in some independent datasets.

### Comparison with previous predictors

To further verify the performance of mRMR + SVM, mRMR + KNN and MRMD + SVM predictor developed in current study, we compared with the existing predictors. Two published studies about finding REOs-based signature for early HCC diagnosis have been completed by Ao et al. and our previous work. In 2018, combining rank difference with majority voting rule, Ao et al. presented a signature by applying 491 HCC samples and 149 CwoHCC samples. This signature, including 19 gene pairs, was chosen from 72 reversal gene pairs. And it yiled the accuracy of 0.9969. In 2020, we identified an early diagnostic signature of HCC from 857 reversal gene pairs on the basis of mRMR and SVM. Using 1091 HCC samples and 242 CwoHCC samples, 11 gene pairs were derived and denoted as the signature, which achieved 1 of accuracy. Due to the difference of training data, a comparison of current results in this paper with existing results in previous studies is an unfair comparison. Therefore, we utilized the same evaluation criteria. To further assessed effectiveness of presented predictors, experimental results in independent datasets were used to perform comparison objectively.

In Table 2, for training set, both mRMR + SVM predictor with 11 gene pairs and mRMR + KNN predictor with 11 gene pairs achieved accuracy of 1, F1-score of 1, as well as the number of gene pairs is the least. Also, MRMD + SVM predictor with 28 gene pairs achieved accuracy of 1, F1-score of 1. As shown in Table 3, for a total of 3057 HCC samples and 84 CwoHCC samples, mRMR + SVM predictor was the best predictor, which yielded AUC of 0.9384, and its accuracy and F1-score were 0.8914 and 0.9351, respectively. In Table 4 and Table S4, for biopsy samples, based on the mRMR + SVM predictor, 96.18% of the 131 HCC samples from 2 datasets (GSE121248, GSE47197) could be correctly identified as HCC. Moreover, 75.26% of the 97 NwHCC samples from 2 datasets (GSE121248 and GSE64041) and all 80 CwHCC samples in GSE54236 were classified as HCC. While, based on MRMD + SVM predictor, all of 131 HCC samples could be correctly identified as HCC, all 97 NwHCC samples and all 80 CwHCC samples were classified as HCC. For surgery samples, 1800 HCC samples from 24 datasets were used to perform evaluation and 657 of them were FFPE HCC samples from 6 datasets. Thus, mRMR + SVM predictor could correctly discriminate 1800 HCC samples and 657 FFPE HCC samples with the sensitivity of 0.8428 and 0.7976, respectively. Also, MRMD + SVM predictor could correctly discriminate 1800 HCC samples and 657 FFPE HCC samples with the sensitivity of 0.9872 and 0.9985, respectively. This result demonstrated that mRMR + SVM predictor and MRMD + SVM predictor had the potential to classify FFPE samples with partial RNA degradation. Moreover, based on mRMR + SVM predictor, 614 out of 741 NwHCC samples from 9 datasets and 229 out of 334 CwHCC samples from 6 datasets were predicted as HCC. While based on MRMD + SVM predictor, all 741 NwHCC samples and all 334 CwHCC samples were predicted as HCC. For RNA-seq data, based on mRMR + SVM predictor, 368 out of 371 HCC samples from TCGA and 11 out of 50 NwHCC tissues were correctly identified as HCC. While based on MRMD + SVM predictor, all 371 HCC samples and all 50 NwHCC tissues were correctly identified as HCC. In addition, 240 out of 243 HCC samples from TCGA were also correctly identified as HCC. While based on MRMD + SVM predictor, all 243 HCC samples were also correctly identified as HCC.

Results in Table S4 displayed the identification of both HCC and its adjacent non-cancer (NwHCC and CwHCC) from CwoHCC by biopsy and surgery samples. For 131 HCC biopsy samples, the sensitivity of proposed mRMR + SVM predictor with 11 gene pairs (18 secreted genes) and MRMD + SVM predictor with 28 gene pairs was 0.7526 and 1, which were higher than Ao’s method (0.6031). The identification ability of proposed mRMR + SVM predictor was also better than Ao’s method in 80 CwHCC samples. Additionally, among these methods, mRMR + SVM predictor and MRMD + SVM predictor displayed the better classification in 657 HCC FFPE samples, 1800 HCC surgery samples (657 HCC FFPE samples were included) and all 1931 HCC samples (1800 HCC surgery samples and 131 HCC biopsy samples were contained). For 657 HCC FFPE samples, the accuracy of Ao’s method, our previous method (11 gene pairs, 2020), proposed mRMR + SVM predictor and MRMD + SVM predictor in this study was 0.172, 0.3973, 0.7976, 0.9985, respectively. For 1800 HCC samples, the accuracy of Ao’s method, our previous method, proposed mRMR + SVM predictor and MRMD + SVM predictor was 0.6639, 0.7656, 0.8428, 0.9872, respectively. For 1931 HCC samples, the accuracy of Ao’s method was 0.6572, the accuracy of our previous method was 0.7815, while the accuracy of the proposed mRMR + SVM predictor and MRMD + SVM predictor could increase to 0.8503 and 0.97, respectively. Above result suggested that mRMR + SVM predictor and MRMD + SVM predictor displayed the better performance when comparing with Ao’s method and our previous method.

In conclusion, methods developed in this paper produced higher accuracy and had superior prediction and diagnosis abilities compared to other published methods, especially for FFPE samples. Therefore, the mRMR + SVM predictor and MRMD + SVM predictor were deemed superior and more suitable predictors for facilitating early HCC diagnosis in clinical practice.

## Conclusions

In this study, we developed eHCC-pred, a machine learning-based predictor for early diagnosis of HCC, using REOs and two feature selection methods (mRMR and MRMD). The eHCC-pred comprised of two machine learning predictors: MRMD + SVM predictor and mRMR + SVM predictor. In the training set consisting of 988 HCC samples and 332 CwoHCC samples, both MRMD + SVM predictor and mRMR + SVM predictor achieved perfect accuracy, F1-score, and AUC values of 1. Subsequently, the performance of these predictors was evaluated on independent datasets comprising 3057 HCC samples and 84 CwoHCC samples. The mRMR + SVM predictor exhibited a higher AUC value (0.9384) compared to the MRMD + SVM predictor (AUC = 0.9278), while the latter attained the highest accuracy of 0.9834 and F1-score of 0.9915. Finally, we compared our results with previous methods in this field. It is important to note that the data preprocessing level of our previous method 2020 (involving 11 gene pairs) is equivalent to the current work. The accuracy of early HCC identification has significantly improved, with a remarkable increase from 78.15 to 97%, based on identical independent datasets. Our approach, eHCC-pred (http://www.dulab.com.cn/eHCC-pred/), is expected to be robustly utilized at an individual level to facilitate early diagnosis of HCC in clinical practice surpassing currently available state-of-the-art techniques.

## Discussion

High accurate and early diagnosis is the key point to hepatocellular carcinoma patients. Current work developed and validated machine learning-based predictors to aid early HCC diagnosis in clinical practice. Among the sixteen predictors, the mRMR + SVM predictor comprising of 11 gene pairs (18 secreted genes) and the MRMD + SVM predictor consisting of 28 gene pairs (34 secreted genes) exhibited superior predictive capability in validation datasets, thereby potentially enhancing the precision of decision-making during HCC diagnosis.

Database PubMed was searched, and GDF15 included in 11 gene pairs (mRMR + SVM predictor) and HTATIP2 included in 28 gene pairs (MRMD + SVM predictor) had been reported to be related to HCC. GDF15 is also called MIC-1 and HTATIP2 is also named TIP30. GDF15^30,31^ and HTATIP2^32^ are effective serum signatures for the diagnosis of HCC. Then, we searched HPA database and found three (GDF15, FCN3, FCN2) of 18 genes (11 gene pairs) were detected by blood-based immunoassay, fourteen (FCN2, GDF15, FCN3, DPT, THY1, ADAMTSL2, ASAH1, C7, DBH, F8, PCOLCE2, PPIC, PRDX4, RPLP2) of 18 secreted genes were detected in plasma by mass spectrometry, and four (FCN2, GDF15, DPT, THY1) of 18 secreted genes were detected in plasma by proximity extension assay (see Table 5). Thus, combining Table 2 with Table 5, we concluded that six (PCOLCE2 & DBH, RPLP2 & FCN3, THY1 & DPT, RPLP2 & ADAMTSL2, PPIC & C7, PRDX4 & C7) of 11 gene pairs were detected in blood. Similarly, for 34 genes (28 gene pairs), three genes (FCN3, CXCL12, FCN2) were detected by blood-based immunoassay, nineteen genes (RPLP2, PCOLCE2, PRDX4, MLEC, DNASE2, THY1, SNTB1, PON2, GLA, TPST2, FCN3, DBH, ADAMTSL2, C7, CXCL12, DPT, FCN2, F8, PAMR1) were detected in plasma by mass spectrometry, and seven genes (THY1, IFNGR1, PON2, CXCL12, DPT, FCN2, PAMR1) were detected in plasma by proximity extension assay (see Table S5). Thus, combing Table S3 with Table S5, we concluded that eighteen (RPLP2 & FCN3, PCOLCE2 & DBH, RPLP2 & ADAMTSL2, PRDX4 & C7, MLEC & CXCL12, KDSR & DPT, EIF2AK1 & CHST4, THY1 & DPT, SNTB1 & SFRP5, KDSR & FCN2, EIF2AK1 & F8, IFNGR1 & CXCL12, PON2 & C7, GLA & CHST4, TPST2 & FCN3, THY1 & PAMR1, THY1 & CHST4, IFNGR1 & C7) of 28 gene pairs were detected in blood.

**Table 5:** Detection of 18 secreted genes in blood from HPA database.

Gene name	Gene description	Blood-based immunoassay	Mass spectrometry	Proximity extension assay
FCN2	Ficolin 2	Detected	Detected	Detected
GDF15	Growth differentiation factor 15	Detected	Detected	Detected
FCN3	Ficolin 3	Detected	Detected	No
DPT	Dermatopontin	No	Detected	Detected
THY1	Thy-1 cell surface antigen	No	Detected	Detected
ADAMTSL2	ADAMTS like 2	No	Detected	No
ASAH1	*N*-Acylsphingosine amidohydrolase 1	No	Detected	No
C7	Complement C7	No	Detected	No
DBH	Dopamine beta-hydroxylase	No	Detected	No
F8	Coagulation factor VIII	No	Detected	No
PCOLCE2	Procollagen C-endopeptidase enhancer 2	No	Detected	No
PPIC	Peptidylprolyl isomerase C	No	Detected	No
PRDX4	Peroxiredoxin 4	No	Detected	No
RPLP2	Ribosomal protein lateral stalk subunit P2	No	Detected	No
CHST4	Carbohydrate sulfotransferase 4	No	No	No
EIF2AK1	Eukaryotic translation initiation factor 2 alpha kinase 1	No	No	No
KDSR	3-Ketodihydrosphingosine reductase	No	No	No
PTPRA	Protein tyrosine phosphatase receptor type A	No	No	No

Over the past decade, there has been a significant growth in the application of machine learning in the field of medicine, particularly in oncology. However, constructing machine learning models often encounters various challenges, including limited data availability, inadequate representation of real-world scenarios in training data, poor data quality encompassing irrelevant features and potential overfitting risks. Our study aimed to effectively train the model by maximizing sample utilization while ensuring a balanced distribution between positive and negative samples. Additionally, we employed feature selection techniques to identify relevant features and eliminate irrelevant ones before evaluating them using independent datasets.

In clinical practice, timely diagnosis is crucial for patients seeking medical attention. Specifically, biopsy and surgery samples obtained from various body tissues were subjected to RNA sequencing and microarray analyses, followed by the generation of TPM or FPKM profiles using the RNA sequencing data. Subsequently, the generated TPM, FPKM, and microarray datasets were employed as inputs for eHCC-pred. By utilizing the eHCC-pred web server, users can access predicted outcomes for patients to facilitate HCC diagnosis.

## Methods

A total of 46 datasets (Table S1) used in this study were collected from three public databases, including GEO (https://www.ncbi.nlm.nih.gov/geo/), ICGC (https://dcc.icgc.org/) and TCGA (https://portal.gdc.cancer.gov/). In total, 5586 tissue samples, consisting of 4045 HCC samples, 416 CwoHCC samples, 334 CwHCC samples and 791 NwHCC samples, were enrolled in this study, the detail of all datasets was shown in Table S1.

The 44 GEO transcriptome datasets contained 3431 HCC samples, 416 CwoHCC samples, 334 CwHCC samples and 741 NwHCC samples. Gene expression profiles from GEO were mainly detected by Affymetrix, Agilent and Illumina platforms. In case of Affymetrix array datasets, raw data (.CEL) files were pre-processed with background correction and normalization by using robust multi-array averaging (RMA) method. In case of Agilent and Illumina array datasets, the processed data (series matrix files) were utilized. Then, the arithmetic mean of multiple probes calculated that correspond to an individual gene for each dataset singly. The RNA-Seq gene expression data of ICGC dataset (the Liver Cancer-RIKEN JP) and TCGA dataset were derived from the ICGC database and TCGA database, respectively. The ICGC transcriptome dataset contained 243 HCC samples, the TCGA transcriptome dataset contained 371 HCC samples and 50 NwHCC samples. Additionally, 2902 secreted genes downloaded from Human Protein Atlas (HPA, https://www.proteinatlas.org/) were also used in this study and it were listed in Table S2.

### Training and validation datasets

In clinical practice, tissue samples were usually obtained through two methods: surgical resection specimens or tissue biopsy samples. In our study, surgery samples and biopsy samples were used. The datasets used to derive the diagnostic signature consisted of a total of 1237 HCC samples, including 141 biopsy samples (D1) and 1096 surgery samples (D2), as well as 416 CwoHCC samples, consisting of 236 biopsy samples (D3) and 180 surgery samples (D4). Subsequently, we randomly divided the dataset (D1, D2, D3, D4) into two subsets: a training set (80%) and a testing set (20%). The training set comprised 988 HCC samples (112 biopsy samples and 876 surgery samples) along with 332 CwoHCC samples (188 biopsy samples and 144 surgery samples). Similarly, the testing set included 249 HCC samples (29 biopsy sample and 220 surgery sample) in addition to 84 CwoHCC samples (48 biopsy and 36 surgery samples). The training set was for the development of the prediction predictors, while testing set and other independent gene-expression datasets (array and RNA-seq) were used as validation datasets for evaluating the performance of the prediction predictors. Validation datasets contained 3057 HCC samples (202 HCC biopsy samples and 2855 HCC surgery samples) and 84 CwoHCC samples (48 CwoHCC biopsy samples and 36 CwoHCC surgery samples).

### Feature construction method

REOs was a feature construction method which has been applied to acquire a dependable and robust signature from gene expression profiling. In case of a gene pair (gene i and gene j), Gi > Gj represented that the expression of gene i was higher than the expression of gene j, Gi < Gj represented that the expression of gene i was lower than the expression of gene j. Stable gene pairs meant that the pattern of Gi > Gj or Gi < Gj was kept in at least 85% samples. One stable gene pairs which kept Gi > Gj in HCC tissues and Gi < Gj in CwoHCC tissues was denoted as a reversal stable gene pair, and then this gene pair would be selected as the candidate REO-based qualitative diagnostic signature. After obtaining reversal gene pairs between HCC and CwoHCC tissues, 2902 secreted genes were used for filtering gene pairs. Next, based on the reversal gene pairs and gene expression profiling, new profiles encoded by 0, 1, and − 1 were generated, where 1 represented Gi < Gj, 0 represented Gi > Gj, − 1 represented other cases (Gi or Gj does not exist), respectively.

### Feature selection method and incremental feature selection

To pick out valid gene pairs for HCC diagnosis, mRMR^21^ and MRMD^22^ algorithms were applied for feature selection. Here, a gene pair was considered as a feature. The principle of mRMR algorithm is simple: to find maximum correlation while removing redundant features, which is equivalent to obtaining a set of “purest” feature subset (features differ greatly from each other and are also highly correlated with the target variable). It is based on information theory and can be computed by mutual information (MI), MI and mRMR were formulated as follows:

1 $$MI(f_{i} ,T) = \int {P(f_{i} ,T)} \ln \left( {\frac{{p(f_{i} ,T)}}{{p(f_{i} )P(T)}}} \right)df_{i} dT,$$

2 $$mRMR = \frac{1}{\left| \Phi \right|}\sum\limits_{{f_{i} \in \Phi }} {MI(f_{i} ,T)} - \frac{1}{{\left| \Phi \right|^{2} }}\sum\limits_{{f_{i} f_{j} \in \Phi }} {MI(f_{i} ,f_{j} )} ,$$

where $f$ represents the vector of feature, $T$ represents disease type, $\Phi$ represents the set of ranked features, $MI(f_{i} ,T)$ represents MI between feature $f_{i}$ and class $T$, and $MI(f_{i} ,f_{j} )$ represents MI between $f_{i}$ and $f_{j}$.

MRMD is to select feature subsets that are strongly correlated with class label and have low redundancy among features. MRMD feature selection method is mainly determined by the following two parts. The first is the correlation between feature and class label. MRMD calculates the correlation between feature and class label by Pearson correlation coefficient. The larger the Pearson correlation coefficient is, the closer the relationship between features and class label is. The second is the redundancy between features. Three distance functions (Euclidean distance, Cosine distance and Tanimoto coefficient) are used to calculate the redundancy between features. And the larger the distance is, the lower the redundancy between features is. More details about MRMD can be found in Zou’s paper^22^. In this study, Cosine distance was used.

Based on the new encoding profiles and two feature selection methods, we obtained a list of ranked gene pairs. Subsequently, using incremental feature selection (IFS) strategy^33^, the optimal gene pairs which could produce the best diagnosis for HCC was chosen from 242 mRMR and MRMD gene pairs.

### Classification through machine learning methods

Machine learning techniques included SVM, KNN, DT, LR, XGBoost, LMT, AdaboostM1 and NB were adopted to establish predictive diagnostic predictors of early HCC. Notably, XGBoost and and NB were performed by using R package “xgboost” and “naivebayes”, respectively. For XGBoost model, The parameters of XGBoost model are nrounds = 25 and objective = “binary:logistic”. Another six classification methods were performed by using R package “RWeka”, the function of SMO, IBk, J48, LR, LMT and AdaBoostM1 was used. And SMO provides a support vector classifier using RBF kernels with a non-default gamma parameter (argument ‘-G’), G = 2. IBk generates a k-nearest neighbors classifier, J48 provides unpruned or pruned C4.5 decision trees, LR produces logistic regression model and LMT carries out “Logistic Model Trees”. The AdaBoost M1 method of Freund and Schapire is implemented by AdaBoostM1 function and decision stumps (trees with a single split only) are used as base learners for AdaBoostM1.

### Performance evaluation of predictors

In the current study, we assessed the performance of our prediction predictors on independent cohorts that include testing set and other independent datasets (array and RNA-seq gene-expression data) obtained from GEO, ICGC and TCGA (see Table S1), which were not used for training. Five popular indexes were calculated to evaluate the diagnostic ability of the gene pair signature for early HCC. They are sensitivity, specificity, accuracy, F1-score and area under receiver operating characteristic curve (AUC).

3 $$\left\{\begin{array}{l}Sensitivity=\frac{TP}{TP+FN}\\ Specificity=\frac{TN}{TN+FP}\\ Accuracy=\frac{TP+TN}{TP+FP+TN+FN}\\ F1{\text{-}}score=\frac{2TP}{2TP+FP+FN}\end{array},\right.$$

where P and N represent the scale of positive (HCC) and negative (CwoHCC) samples, respectively. T and F represent sets of true and false predicted results, respectively.

Receiver operating characteristic (ROC) curve is a tool to analyze the classification performance of binary classification model. The ROC space defines the false positive rate (FPR) as the X-axis and the true positive rate (TPR) as the Y-axis. And the area under the curve is calculated to compute AUC value. AUC is used to measure ranking ability. AUC with 95% CI (confidence intervals) is the Area Under the ROC Curve and a probability value. The larger the AUC is, the better the classification performance is.

### Supplementary Information

Supplementary Tables.

## Data Availability

The datasets generated and/or analysed during the current study are available in the GEO repository (https://www.ncbi.nlm.nih.gov/geo/), ICGC repository (https://dcc.icgc.org/) and TCGA repository (https://portal.gdc.cancer.gov/). Additionally, 2902 secreted genes downloaded from Human Protein Atlas (HPA, https://www.proteinatlas.org/).
